# Statistical evaluation of proxies for estimating the rainfall erosivity factor

**DOI:** 10.1038/s41598-022-15271-x

**Published:** 2022-07-15

**Authors:** Xiaoqing Ma, Mingguo Zheng

**Affiliations:** 1 National-Regional Joint Engineering Research Center for Soil Pollution Control and Remediation in South China, Guangdong Key Laboratory of Integrated Agro-environmental Pollution Control and Management, Guangdong Engineering Research Center for Non-point Source Pollution Control, Institute of Eco-environmental and Soil Sciences, Guangdong Academy of Sciences, Guangzhou, 510650 People’s Republic of China; 2 Key Laboratory of Water Cycle and Related Land Surface Processes, Institute of Geographic Sciences & Natural Resources Research, Chinese Academic of Sciences, Beijing, 100101 People’s Republic of China; 3 College of Resources and Environment, University of Chinese Academy of Sciences, Beijing, 100101 People’s Republic of China; 4 International Institute of Soil and Water Conservation, Meizhou, 514000 People’s Republic of China

**Keywords:** Environmental impact, Hydrology, Hydrology

## Abstract

Considering the high-temporal-resolution rainfall data requirements for calculating the Rainfall Erosivity factor (that is, the *R*-factor), studies have developed a large number of proxies for the *R*-factor (*PR*). This study aims to evaluate 15 widely used proxies, which were developed in various countries using daily, monthly, or yearly rainfall data, in terms of correlation and statistical equality with the *R*-factor by using the 6-min pluviographic data from 28 stations in Australia. Meng’s test was applied to rank the correlations. Although the Meng’s test indicated that the correlation between Rainfall Erosivity (*R*) and Rainfall Erosivity calculated by the proxy model (*PR*) generally increased with a finer time resolution of the rainfall data (in the order of year, month, and day), the 15 *PR*s under examination were all highly correlated with *R* (*r* > 0.62, *p* < 0.004), implying that all of them can be reasonably used as an *R* predictor. A direct estimation of the *R*-factor using *PRs* produced a mean relative error (*MRE*), root mean square error (*RMSE*), and Nash–Sutcliffe efficiency coefficient (*NSE*) with a mean of 50.0%, 1392 MJ mm ha^−1^ h^−1^ a^−1^, and 0.17, respectively. The linear calibrations improved the accuracy of the estimation and produced an *MRE*, *RMSE*, and *NSE* with a mean of 36.0%, 887 MJ mm ha^−1^ h^−1^ a^−1^, and 0.70, respectively. Finally, suitable proxies for instances where only daily, monthly, or yearly rainfall data are available were recommended.

## Introduction

Soil erosion can lead to land degradation and environmental deterioration^1–3^. Eroded sediments also have negative off-site effects, such as impaired water quality, altered aquatic habitats, limited channel navigability, reduced hydroelectric equipment lifespan, and increased flood risk^4–6^. Rainfall is a driver of water erosion. Unlike other natural factors affecting erosion, such as topography and vegetation, rainfall is less subject to human alteration or control. Rainfall thus represents an environmental contributor to soil loss and is of great importance in assessing or predicting soil erosion intensity^7^.

Rainfall Erosivity refers to the potential of a storm to erode the soil. The universal soil loss equation (USLE)^8,9^ and the revised universal soil loss equation (RUSLE, RUSLE2)^10,11^, the most widely used models for predicting soil erosion, applied equations (Eqs. 1 and 2) to quantify the average annual Rainfall Erosivity, *R* (MJ mm ha^−1^ h^−1^ a^−1^). The discrepancy is reflected in the algorithm of the estimated unit rainfall kinetic energy (*e*_*r*_, MJ mm^−1^ ha^−1^), and Eqs. (3–5) are utilized by USLE, RUSLE, and RUSLE2, respectively.

1 $$R = \frac{1}{n}\mathop \sum \limits_{j = 1}^{n} \mathop \sum \limits_{k = 1}^{m} \left( {EI_{30} } \right)_{k}$$

2 $$E = \left( {\mathop \sum \limits_{r = 1}^{n} \left( {e_{r} \cdot P_{r} } \right)} \right)$$

3 $$e_{r} = \left\{ {\begin{array}{*{20}l} {0.119 + 0.0873\log i_{r} \, i_{r} \le 76.2\,{\text{mm/h}}} \hfill \\ {0.283\,i_{r} > 76.2\,{\text{mm/h}}} \hfill \\ \end{array} } \right.$$

4 $$e_{r} = 0.29\left[ {1 - 0.72exp\left( { - 0.05i_{r} } \right)} \right]$$

5 $$e_{r} = 0.29\left[ {1 - 0.72exp\left( { - 0.082i_{r} } \right)} \right]$$

where *n* is the number of years of the record, *m* is the number of erosive events in each year, *EI*_*30*_ is the Rainfall Erosivity index of a single event, *E* is the total rainfall energy (MJ ha^−1^), *I*_*30*_ is the maximum 30 min rainfall intensity (mm h^−1^), and *P*_*r*_ and *i*_*r*_ are the rainfall amount (mm) and rainfall intensity of the *r*th time interval (mm h^−1^), respectively.

Comparing the three equations for the *R*-factor, the results indicated that the RUSLE significantly underestimates the *R* value^12–15^. Nearing et al.^14^ stated that the *R* value calculated by the RUSLE was 14% lower than that of the USLE and RUSLE2; however, the USLE and RUSLE2 results showed resemblances based on data from 56 stations from three countries: China, Italy, and the US. Studies show that the RUSLE used to estimate the *R*-factor is based on a conceptual mistake that results in underestimation of the *R* value; hence, Eq. (4) (RUSLE) should not be used for *R* calculation and Eq. (5) (RUSLE2) is recommended to be used instead^14^. The RUSLE2 is commonly utilized by the US government for conservation planning^16^ and has been used in recent studies on Rainfall Erosivity^17,18^.

The *EI*_30_ index is a standard algorithm for calculating Rainfall Erosivity. However, the algorithm requires pluviographic records at < 15 min intervals^9^; moreover, the record period should be longer than 20 years for the purpose of containing dry and wet climatic regimes^19^. Such rainfall data are often unavailable, particularly in developing countries^20^. In addition, the algorithm is complicated and often laborious and time-consuming^21^.

Considering the limited data availability and tedious process, many proxies for the *R*-factor (*PR*) have been developed, using daily, monthly, and annual data instead of pluviographic data. Table 1 lists 15 proxies that have been extensively applied in the literature, three of which have been developed in the US, two in Spain, two in Malaysia, five in China, two in Australia, and one in Honduras.

**Table 1:** Established proxies for the *R*-factor.

Rainfall data	Equations	Countries where *PRi* was developed	References
Annual	*PR*_1_ = 0.04830*MAP*^1.610^ *P* < 850 mm *PR*_1_ = 587.8–1.219 *MAP* + 0.004105 *MAP *^2^ *P* > 850 mm	US	^38^
	*PR*_2_ = 699.3 + 7.0001 *MAP*-2.7190*H*	Honduras	^39^
Monthly	$PR_{3} = 9.8 \times \mathop \sum \limits_{i = 1}^{n} AR_{i} = \mathop \sum \limits_{i = 1}^{n} \mathop \sum \limits_{j = 1}^{12} 1.735 \times 10^{{\left[ {1.5log\left( {\frac{{p_{j}^{2} }}{{p_{a} }}} \right) - 0.8188} \right]}}$	US	^40^
	*PR*_4_ = 0.07397 *MFI*^1.847^ MFI < 55 mm *PR*_4_ = 95.77–6.081*MFI* + 0.4770*MFI*^2^ MFI > 55 mm $MFI = \mathop \sum \limits_{i = 1}^{12} \frac{{p_{i}^{2} }}{MAP}$	US	^38^
	*PR*_5_ = 21.56*MFI*^0.927^	Spain	^7^
	*PR*_6_ = 25.1*F* $F = \frac{{p^{2} }}{MAP}$	Spain	^36^
	*PR*_7_ = 0.3589*MFI*^1.9462^	China	^41^
	$PR_{8} = 10 \times \mathop \sum \limits_{j = 1}^{12} 0.0125p_{j}^{1.6295}$	China	^42^
Daily	$PR_{9} = \mathop \sum \limits_{i = 1}^{n} \mathop \sum \limits_{j = 1}^{12} MR_{j} = \mathop \sum \limits_{i = 1}^{n} \mathop \sum \limits_{j = 1}^{12} 6.97\left( {rain_{10} } \right)_{j} - 11.23\left( {day_{10} } \right)_{j}$	Malaysia	^32^
	$PR_{10} = \mathop \sum \limits_{i = 1}^{n} \mathop \sum \limits_{j = 1}^{12} MR = \mathop \sum \limits_{i = 1}^{n} \mathop \sum \limits_{j = 1}^{12} 0.266(rain_{10} )_{j}^{2.071} (day_{10} )_{j}^{ - 1.367}$	Malaysia	^32^
	$PR_{11} = \mathop \sum \limits_{i = 1}^{n} \mathop \sum \limits_{k = 1}^{24} \left( {HMR} \right)_{k} = \mathop \sum \limits_{a = 1}^{n} \mathop \sum \limits_{k = 1}^{24} \alpha \mathop \sum \limits_{m = 1}^{n} \left( {P_{d \ge 12} } \right)_{m}^{\beta }$ $\alpha = 21.586\beta^{ - 7.1891}$ $\beta = 0.8363 + \frac{18.144}{{P_{d12} }} + \frac{24.455}{{P_{y12} }}$	China	^43^
	$PR_{12} = \mathop \sum \limits_{i = 1}^{n} \mathop \sum \limits_{k = 1}^{24} \left( {HMR} \right)_{k} = \mathop \sum \limits_{a = 1}^{n} \mathop \sum \limits_{k = 1}^{24} \alpha \mathop \sum \limits_{m = 1}^{n} \left( {P_{d \ge 12} } \right)_{m}^{\beta }$ $\alpha = 10^{{2.124 - 1.495\beta + 0.00214P_{dmax} }}$ $\beta = 0.8363 + \frac{18.144}{{P_{d12} }} + \frac{24.455}{{P_{y12} }}$	China	^44^
	$PR_{13} = \mathop \sum \limits_{i = 1}^{n} \mathop \sum \limits_{k = 1}^{24} \left( {HMR} \right)_{k} = \mathop \sum \limits_{a = 1}^{n} \mathop \sum \limits_{k = 1}^{24} \alpha \mathop \sum \limits_{m = 1}^{n} \left( {P_{d \ge 12} } \right)_{m}^{1.7265}$ $$\alpha = \left\{ {\begin{array}{*{20}c} {{0}{\text{.3937 warm season (10{-}12months, 1{-}4months)}}} \\ {0.3101{ }cold season(5{-}9 {\text{months)}}} \\ \end{array} } \right.$$	China	^45^
	$$PR_{14} = \mathop \sum \limits_{i = 1}^{n} \mathop \sum \limits_{j = 1}^{12} MR_{j} = \mathop \sum \limits_{i = 1}^{n} \mathop \sum \limits_{j = 1}^{12} \alpha \left[ {1 + \eta cos\left( {2\pi fi - \omega } \right)} \right]\mathop \sum \limits_{m = 1}^{n} \left( {P_{d \ge 12.7} } \right)_{m}^{\beta }$$$$f = \frac{1}{12};\omega = \frac{\pi }{6};\beta = 1.49;\eta = 0.29$$ $$\alpha = 0.359\left[ {1 + 0.0989exp\left( {3.26S/MAP} \right)} \right]$$	Australia	^46^
	$PR_{15} = \mathop \sum \limits_{i = 1}^{n} \mathop \sum \limits_{j = 1}^{12} MR_{j} = \mathop \sum \limits_{i = 1}^{n} \mathop \sum \limits_{j = 1}^{12} \alpha \left[ {1 + \eta cos\left( {2\pi fi - \omega } \right)} \right]\mathop \sum \limits_{m = 1}^{n} \left( {P_{d \ge 0} } \right)_{m}^{\beta }$ $f = \frac{1}{12};\omega = \frac{\pi }{6};\eta = 0.3$ ${\upbeta } = 1.02 - 0.0209{\text{L}}$ $\alpha_{0} = 1.05 \times 10^{{\left( {2.08 - 1.58\beta } \right)}}$ $\alpha /\alpha_{0} = 2.349 + 0.04040L - 0.0002684H$	Australia	^47^

*PR*_*i*_ is the *i*th proxy for the *R*-factor.

*AR*_*i*_, *MR*_*j*_, and *HMR*_*k*_ (MJ mm ha^−1^ h^−1^ a^−1^) are the Rainfall Erosivity in the *i*th year, *j*th month, and *k*th half-month, respectively.

*p*_*j*_, *p*_a_, *p*_*i*_, and *p* are the rainfall in month *j*, annual rainfall, average rainfall in month *i*, and highest monthly rainfall, respectively.

*rain*_10_ (mm) represents monthly rainfall exceeding 10 mm for days, and *day*_10_ represents the monthly rainfall exceeding 10 mm for days.

(*P*_*d* > 12.7(12, 0)_)_*m*_ (mm) represents daily rainfall of ≥ 12.7 (12, 0) mm on the *m*th day.

*P*_*dmax*_ (mm) represents the maximum daily rainfall in an average year.

*H* (mm) and *L* are the altitude and latitude in decimal degrees of the stations, respectively.

*S* (mm) is the average rainfall for the summer half of the year (November–April, Bureau of Meteorology, 1989).

*MAP* (mm) is the mean annual precipitation.

α, β, and η are regional constants.

*F* and *MFI* represent the Fournier index^48^ and the modified Fournier index^35^, respectively.

To assess candidate proxies, studies have often used the Nash–Sutcliffe efficiency coefficient^22^ and the relative error to evaluate the discrepancies between the *R*-factor and the proxies^7,23–27^. However, the most suitable proxy was determined simply through a direct comparison of the obtained Nash–Sutcliffe coefficient and the relative error, which vary with the collected sample data and are random variables. Because the comparison among random variables should be done in the framework of statistics, a direct comparison of the obtained Nash–Sutcliffe coefficient or relative error is questionable.

To the best of our knowledge, no studies have conducted statistical evaluations of the established *PR*. The objective of this study was to statistically evaluate the 15 *R*-proxies listed in Table 1 using rainfall data from Australia. We ranked the correlations between the proxies and *R*-factor using Meng’s test. Based on the statistical evaluations, we recommended proxies for instances where only daily, monthly, or yearly rainfall data are available.

## Methodology

### Data source

We used 6-min pluviographic rainfall data from 28 stations in Australia (Table 2), which were provided by the Australian Government Bureau of Meteorology (http://www.bom.gov.au). Australia has a diverse climate, including tropical monsoon climate in the northern part, Mediterranean climate in the southwestern corner of the country, oceanic and humid subtropical climate in the southeastern part, and arid to semi-arid climate in the interior mainland. Rainfall has high spatial variation, ranging from < 50 mm to > 3000 mm, with a mean of 465 mm (Table 2). Detailed information on the selected stations is listed in Table 2, including location information, length of record, mean annual precipitation (*MAP*), and *R* value. The selected stations spanned from tropical to subtropical and temperate, and from humid to semi-arid and arid regions, with a *MAP* varying from 195 to 1236.2 mm. Most of the selected stations (22 out of the 28 stations) had a *MAP* ranging between 400 and 800 mm because soil erosion is rather limited in very humid or very dry areas. The missing rainfall data for each station varied, and for the purpose of the quality of the rainfall data, the maximum missing rate of each year should not be greater than 90% for each station. Hence, the data for the selected period were unequal. Rainfall data from the 28 stations were for the period 1960–2011, and the data periods ranged from 20 to 31 years, with a mean of 23 years for the 28 selected stations.

**Table 2:** Detailed information on the 28 selected stations.

Station No. and name	Alt. (m)	Lat. (ºS)	Long. (ºE)	Length of record (years)	Annual Rainfall (mm)	Rainfall Erosivity (MJ mm ha^−1^ h^−1^ a^−1^)
03,003 BROOME AIRPORT	7.4	− 17.95	122.24	26 1960–1985	629.4	4950
11,004 FORREST AERO	156.0	− 30.84	128.11	31 1964–1994	195.3	280
15,085 BRUNETTE DOWNS	218.0	− 18.64	135.95	23 1988–2011	427.0	2082
15,548 RABBIT FLAT	340.0	− 20.19	130.02	26 1970–1995	410.4	1859
15,590 ALICE SPRINGS AIRPORT	546.0	− 23.80	133.89	26 1970–1995	296.4	1051
22,816 PARNDANA (ALLANDALE)	232.0	− 35.77	137.03	24 1986–2000	745.3	887
28,004 PALMERVILLE	203.8	− 16.00	144.08	24 1975–1998	984.4	6358
30,018 GEORGETOWN POST OFFICE	291.7	− 18.29	143.55	22 1979–2005	675.3	4339
35,029 GILIGULGUL	318.0	− 26.36	150.05	23 1974–1996	570.6	2288
35,069 TAMBO POST OFFICE	395.1	− 24.88	146.26	20 1985–2004	504.4	1765
35,098 EMERALD DPI TOWN SITE	180.0	− 23.50	148.15	20 1963–1982	611.1	3418
36,031 LONGREACH AERO	192.2	− 23.44	144.28	23 1970–1992	426.3	1728
39,090 THEODORE DPI	142.0	− 24.95	150.07	24 1955–1978	662.7	3079
40,223 BRISBANE AERO	4.0	− 27.42	153.11	23 1955–1977	1236.2	5774
41,139 WYAGA STATION	251.0	− 28.17	150.65	22 1990–2011	513.2	2167
41,521 GOONDIWINDI AIRPORT	217.6	− 28.52	150.33	20 1992–2011	554.3	2317
43,015 INJUNE POST OFFICE	390.0	− 25.84	148.57	24 1984–2007	552.4	2394
43,053 ST GEORGE	200.0	− 28.05	148.56	23 1970–1992	445.9	1808
43,091 ROMA AIRPORT	307.4	− 26.55	148.77	20 1993–2011	492.6	2102
53,048 MOREE COMPARISON	212.1	− 29.48	149.84	25 1970–1994	587.1	1664
62,020 BYLONG (MONTORO)	400.0	− 32.50	150.03	21 1970–1990	644.8	1481
65,035 WELLINGTON RESEARCH CENTRE	390.0	− 32.51	148.97	22 1962–1984	583.1	1317
72,023 HUME RESERVOIR	184.0	− 36.10	147.03	25 1980–2004	670.6	1231
76,031 MILDURA AIRPORT	50.0	− 34.24	142.09	26 1955–1980	298.1	449
80,109 COBRAM (GOULBURN MURRAY)	113.0	− 35.91	145.64	22 1980–2004	448.4	624
82,042 STRATHBOGIE	502.0	− 36.85	145.73	30 1980–2002	964.7	1848
84,121 ORBOST SRWSC	45.4	− 37.70	148.46	21 1973–1993	799.2	1275
86,038 ESSENDON AIRPORT	78.4	− 37.73	144.91	20 1952–1971	607.2	736

### Calculation of the R-factor using the RUSLE2 and its proxies

The *R* value is generally underestimated using the RUSLE2, as discussed in the Introduction section. Hence, the *R*-factor was calculated using Eqs. (1), (2), and (5) (RUSLE2), and the 15 *PR*s (the proxies for the *R*-factor; Table 1) were calculated using the models listed in Table 1. In the calculation, we defined two consecutive rainfall events as having a time interval longer than 6 h, as suggested by Wischmeier and Smith^9^. The < 12.7 mm rainfall events, which have limited erosivity, were excluded by the USLE; in contrast, the RUSLE used all rainfall events while calculating Rainfall Erosivity. In Australia, exclusion of the < 12.7 mm rainfall events had negligible consequences. However, in the areas with a < 400 mm *MAP*, where all rainfall events should be included in calculating Rainfall Erosivity^28^. Therefore, we used all rainfall events to calculate the *R* values.

### Using Meng’s test to rank correlations

The correlation coefficient indicates agreement between two variables, and we applied the Pearson's correlation coefficient in this study. The strength of the correlation between *R* and *PR*_*i*_ (the *i*th proxy) is very useful for evaluating the applicability of a *PR*_*i*_. Methods to compare correlations differ depending on whether the correlations are dependent or independent. The correlations between *R* and *PR*_*i*_ are dependent because they have a common dependent variable, that is, the *R*-factor. This study used Meng’s test^29^ to compare correlations between *R* and *PR*_*i*_. Meng’s test was designed to compare the correlations and has been extensively applied in psychological research. Zheng and Chen^30^ used this test to determine the most accurate Rainfall Erosivity indices for single storms on the Chinese Loess Plateau.

Meng et al.^29^ designed a *Z*-test to compare two correlations (*r*_1_ and *r*_2_) that have a common dependent variable. The equations are as follows:

6 $$Z = \left( {z_{r1} - z_{r2} } \right)\sqrt {\frac{N - 3}{{2\left( {1 - r_{x} } \right)h}}}$$

7 $$h = \frac{{1 - f\overline{{r_{i}^{2} }} }}{{1 - \overline{{r_{i}^{2} }} }}$$

8 $$f = \frac{{1 - r_{x} }}{{2\left( {1 - \overline{{r_{i}^{2} }} } \right)}}$$

where *z*_*ri*_ is the Fisher z-transformed value for *r*_*i*_ (i = 1 or 2), ${\text{z}}_{{{\text{ri}}}} = \frac{1}{2}\ln \frac{{1 + {\text{r}}_{{\text{i}}} }}{{1 - {\text{r}}_{{\text{i}}} }}$ ${\text{z}}_{{{\text{ri}}}} = \frac{1}{2}\ln \frac{{1 + {\text{r}}_{{\text{i}}} }}{{1 - {\text{r}}_{{\text{i}}} }}$, *N* is the sample size, *r*_x_ is the correlation between the two independent variables, $\overline{{r_{i}^{2} }}$ = (*r*_1_^2^ + *r*_2_^2^) / 2, and *f* should be set to 1 if it is greater than 1.

To compare *k* correlations with a common dependent variable (*r*_1_, *r*_2_,…, *r*_*i*_,…, *r*_*k*_; *k* > 2), Meng’s test becomes

9 $$Z = r_{{\lambda z_{r} }} \sqrt {\chi^{2} \left( {k - 1} \right)}$$

where $r_{{\lambda z_{r} }}$ represents the correlation coefficient between *z*_*ri*_ and λ_*i*_, *z*_*ri*_ is the Fisher z-transformed value for *r*_*i*_, and λ_*i*_ is the contrast weight assigned to each *z*_*ri*_ and sums to zero; for example, λ_*i*_ should be − 3, 1, 1, and 1 if *r*_1_ is under examination and *k* = 4. ${\upchi }^{2} \left( {k - 1} \right)$ represents χ^2^ distributed with *k*-1 degrees of freedom.

10 $$\chi^{2} \left( {k - 1} \right) = \frac{{\left( {N - 3} \right)\mathop \sum \nolimits_{i = 1}^{k} \left( {Z_{ri} - \overline{Z}_{r} } \right)^{2} }}{{\left( {1 - r_{x} } \right)h}}$$

where *N* is the sample size, $\overline{{z_{r} }}$ is the mean of *z*_*ri*_, *r*_x_ is the median intercorrelation among the *k* independent variables, and $\overline{{r_{i}^{2} }}$ is the mean of *r*_*i*_^2^.

By comparing *r*_*i*_ and the average of the other *k*-1 correlation coefficients, Meng’s test determines whether *r*_*i*_ is a contrast in the two-tailed test. In the case of the one-tailed test, Meng’s test determines whether *r*_*i*_ is a less- or better-correlated contrast. This study used a stepwise Meng’s test procedure to rank the correlations between *R* and *PR*_*i*_*.* We first labeled each of the correlations between *R* and *PR* as better-correlated contrast, less-correlated contrast, or non-contrast by means of the one-tailed Meng’s *Z*-test, whereby the correlations under examination were grouped into two classes: the less-correlated class and the other class. Then, we repeated the procedure for each of the two classes, and the procedure did not terminate until no contrast was detected among any subclass. The significance level was set at 0.05. Bonferroni correction applies when several bivariate tests are performed simultaneously. We do not use Eq. (9) multiple times simultaneously, so that the Bonferroni correction is not necessary. In this way, we ranked the correlation between *R* and *PR*.

In addition to the correlation coefficient, we also used the *MRE* (%), the *RMSE* (MJ mm ha^−1^ h^−1^ a^−1^), and *NSE* to assess the performance of the regression equation. The *MRE*, *RMSE*, and *NSE* are defined as follows:

11 $$MRE = \mathop \sum \limits_{i = 1}^{n} 100 \times \left| {R_{i} - PR_{i} } \right|/R_{i} /n$$

12 $$RMSE = \sqrt {\mathop \sum \limits_{i = 1}^{n} \left( {PR_{i} - R_{i} } \right)^{2} /n}$$

13 $$NSE = 1 - \mathop \sum \limits_{i = 1}^{n} \left( {R_{i} - PR_{i} } \right)^{2} /\mathop \sum \limits_{i = 1}^{n} \left( {R_{i} - \overline{R}} \right)$$

where *R*_*i*_ and *PR*_*i*_ are the* R* and *PR* values of the *i*th station, respectively, *n* is the number of stations, and ${\text{R}}({-\!\!-})$ is the average value of *R*_*i*_.

## Results and discussion

### Calculation results and R and PR deviations

**Figure 1:**
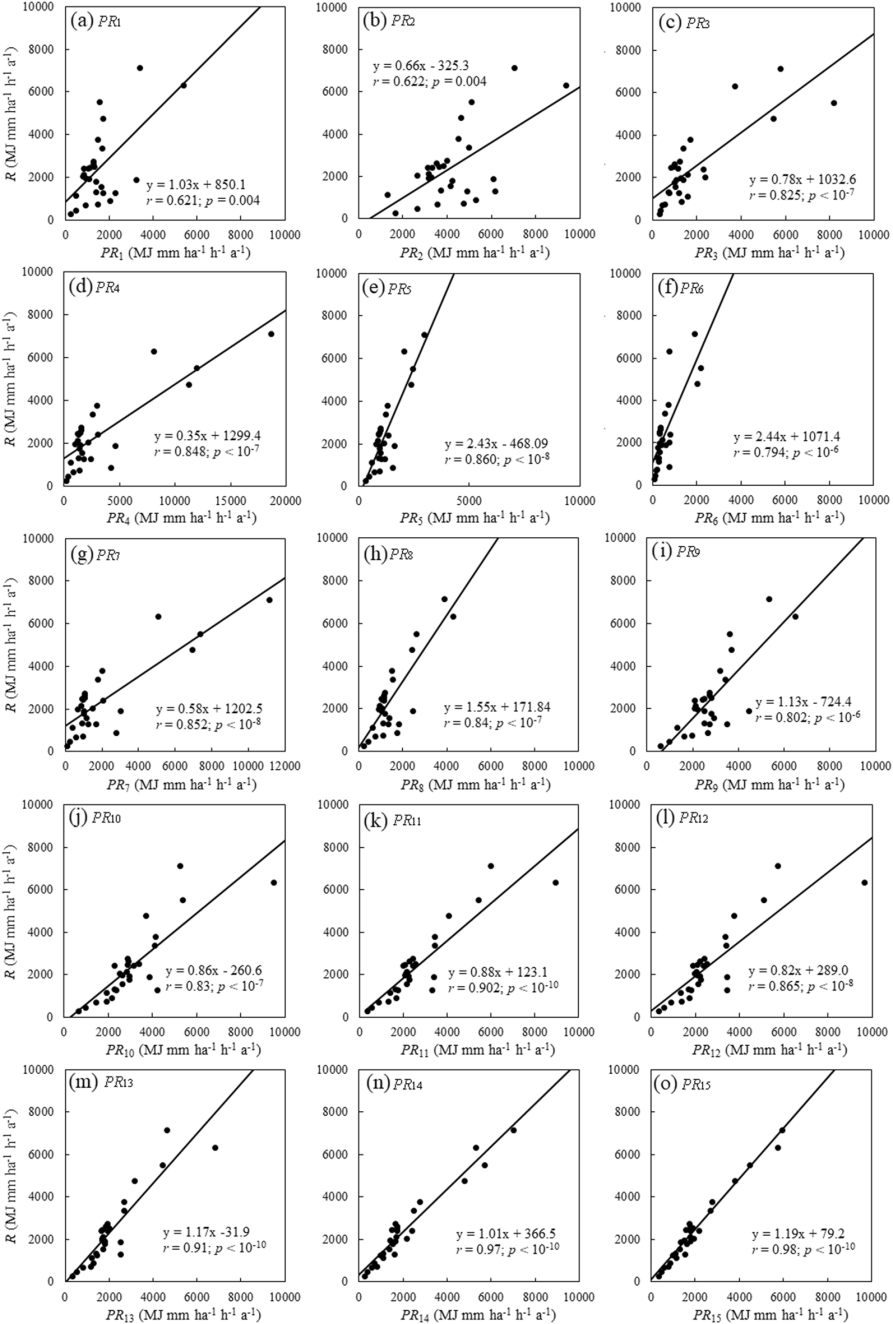
Linear regression between *R* and *PR.*

The calculated *R*-factor of the 28 stations varied between 290.8 MJ mm ha^−1^ h^−1^ a^−1^ and 7153.7 MJ mm ha^−1^ h^−1^ a^−1^ with a mean of 2424.1 MJ mm ha^−1^ h^−1^ a^−1^. As shown in Fig. 1, all examined proxies were surprisingly found to be highly correlated with the *R*-factor (*r* > 0.62, *p* < 0.004), implying that all of them can be reasonably used as a predictor of the *R*-factor in Australia. This finding is at odds with the common notion that regional differences in physiographic settings prevent a model of the *R*-factor from application to other areas. The reason might be that the *PR* models selected were established in different regions and developed by the *R* value calculated by the USLE or RUSLE method. The *PR* value is in fact an approximation of the *R*-factor calculation of the USLE or RUSLE. Therefore, the *PR* value is significantly related to the *R* value, and the difference between *PR* and *R* reflects the correction coefficient, which is the regional difference, and therefore all *PR* values were highly correlated with the *R* values.

The linear regression equation in Fig. 1 can be applied to modify the deviation between the *PR* and *R*. Table 3 lists the *RMSE*, *MRE*, and *NSE* for the estimation of *R* values that directly used *PR* and modified *PR.* The direct estimation showed high deviation, the *MRE* ranged from 18.3 to 153.0%, the *RMSE* ranged from 561 to 2279 MJ mm ha^−1^ h^−1^ a^−1^, and the *NSE* ranged from − 2.1 to 0.89. The revised calculations, as shown in Table 3, indicated the *MRE* ranged from 13.1 to 77.4%, the *RMSE* ranged from 302 to 1482 MJ mm ha^−1^ h^−1^ a^−1^, and the *NSE* ranged from 0.23 0.97. The *RMSE* (*p* = 0.002) and the *MRE* (*p* = 0.01) were notably reduced, and the *NSE* (*p* = 0.009) significantly increased under the correction, which further proved that the error was string-reduced when the modified *PR* was used. However, not all *PR*s showed similar expression, such as the *RMSE*, *MRE,* and *NSE* of *PR*_11_, *PR*_12_, *PR*_13_, and *PR*_14_, which changed slightly after calibration.

**Table 3:** *MRE*, *RMSE* and *NSE* of the selected *PR*s in estimating the *R* value.

*PR*_*i*_	Direct estimation			Modified estimation		
	*MRE*	*RMSE*	*NSE*	*MRE*	*RMSE*	*NSE*
	(%)	(MJ mm ha^−1^ h^−1^ a^−1^)	/	(%)	(MJ mm ha^−1^ h^−1^ a^−1^)	/
*PR*_1_	50.3	1599	0.10	56.5	1323	0.39
*PR*_2_	153.0	2279	− 0.82	77.4	1482	0.23
*PR*_3_	37.4	1214	0.48	37.6	955	0.68
*PR*_4_	62.4	2971	− 2.10	39.8	896	0.72
*PR*_5_	44.8	1732	− 0.05	33.6	862	0.74
*PR*_6_	77.1	2275	− 0.82	36.6	1027	0.63
*PR*_7_	50.7	1405	0.31	38.6	884	0.73
*PR*_8_	43.2	1433	0.28	40.8	927	0.70
*PR*_9_	54.4	1080	0.59	39.2	1008	0.64
*PR*_10_	59.6	1197	0.50	28.9	940	0.69
*PR*_11_	26.1	787	0.78	25.0	730	0.81
*PR*_12_	29.6	926	0.70	30.1	846	0.75
*PR*_13_	23.6	812	0.77	24.8	708	0.82
*PR*_14_	19.6	561	0.89	18.6	410	0.94
*PR*_15_	18.3	609	0.87	13.1	302	0.97
Average	50.0	1392	0.17	36.0	887	0.70

### Correlation comparison between R and PR_S_ using Meng’s test

**Figure 2:**
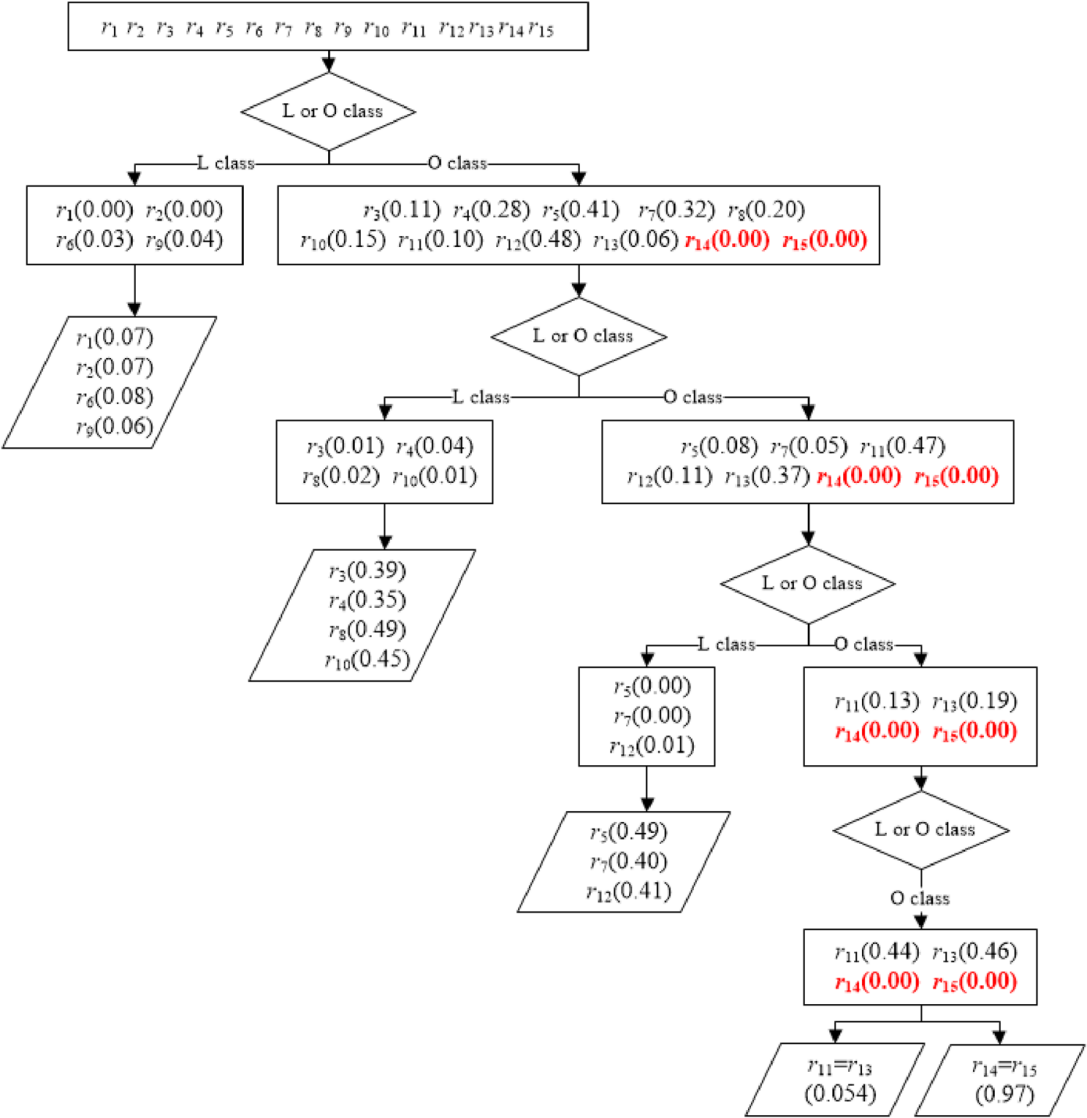
Results of comparing correlations between *R* and *PR* by the stepwise Meng’s test procedure. The numbers within parentheses are the *p* values resulting from the Meng’s tests, the L class represents the less-correlated class, and the O class represents the classes other than those in the L class, including the non-contrast and the better-correlated contrast. Bold and red texts denote the better-than-average correlated.

According to the step-by-step test method designed in this study, the flow chart is shown in Fig. 2, Eq. (9) divided the correlation coefficients of 15 *PR*s and *R* into two classes: L_1_ (*r*_1_, *r*_2_, *r*_6_, *r*_9_), and O_1_ (*r*_3_, *r*_4_, *r*_5_, *r*_7_, *r*_8_, *r*_10_, *r*_11_, *r*_12_, *r*_13_, *r*_14_, *r*_15_), and no significant difference was observed in the L_1_ class (*p* > 0.06). The correlation coefficients in the O_1_ class were separated into L_2_ (*r*_3_, *r*_4_, *r*_8_, *r*_10_) and O_2_ (*r*_5_, *r*_7_, *r*_11_, *r*_12_, *r*_13_, *r*_14_, *r*_15_) classes, and no contrast was detected in the L_2_ class (*p* > 0.35). The correlation coefficients in the O_2_ class were divided into L_3_ (*r*_5_, *r*_7_, *r*_12_) and O_3_ (*r*_11_, *r*_13_, *r*_14_, *r*_15_) classes, and the coefficients in the L_3_ class showed statistical equality under Meng’s test. The correlation coefficients of *PR*_14_ and *PR*_15_ were notably greater than those of *r*_11_ and *r*_13,_ and further statistical tests indicated no significant differences between *r*_11_ and *r*_13,_ *r*_14_, and *r*_15_, respectively. After four rounds (Fig. 2), the stepwise Meng’s test procedure ranked the correlation between *PR*_i_ and *R*, *r*_*i*_, as follows:

14 $$r_{1 \, } = r_{2} = r_{6} = r_{9} < r_{3} = r_{4} = r_{8} = r_{10} < r_{5} = r_{7} = r_{12} < r_{11} = r_{13} < r_{14} = r_{15}$$

As expected, the correlation generally became stronger with finer time resolution of the rainfall data, and the daily proxies were stronger correlated with *R* than the monthly ones, which in turn were stronger correlated with *R* than the yearly ones. The *MRE*, *RMSE*, and *NSE* (Table 3) showed a very similar rank to the correlation in Eq. (14).

### Recommending the PR for rainfall data with different resolutions

Two yearly proxies, *PR*_1_ and *PR*_2_ showed strong correlation with *R* (Fig. 1a,b). The t-test indicated that the coefficient of *PR*_1_ was equal to 1, and the constant term of *PR*_1_ was, significantly, equal to 0. It showed that the *R* value in Australia can be directly estimated by *PR*_1_, and Table 3 shows that no striking variation was observed among the *MRE*, *RMSE* and *NSE* when estimating the *R* value by revised *PR*_1_. The coefficient and constant term of *PR*_2_ were significantly unequal to 1 and 0, respectively. It showed that the estimation of the *R* value in Australia directly by *PR*_2_ would result in a large discrepancy. Based on Eq. (14), no statistical difference was observed between *r*_1_ and *r*_2_. Hence, we suggest the use of *PR*_1_ and revised *PR*_2_ as predictors of the *R*-factor when only yearly rainfall data are available.

**Figure 3:**
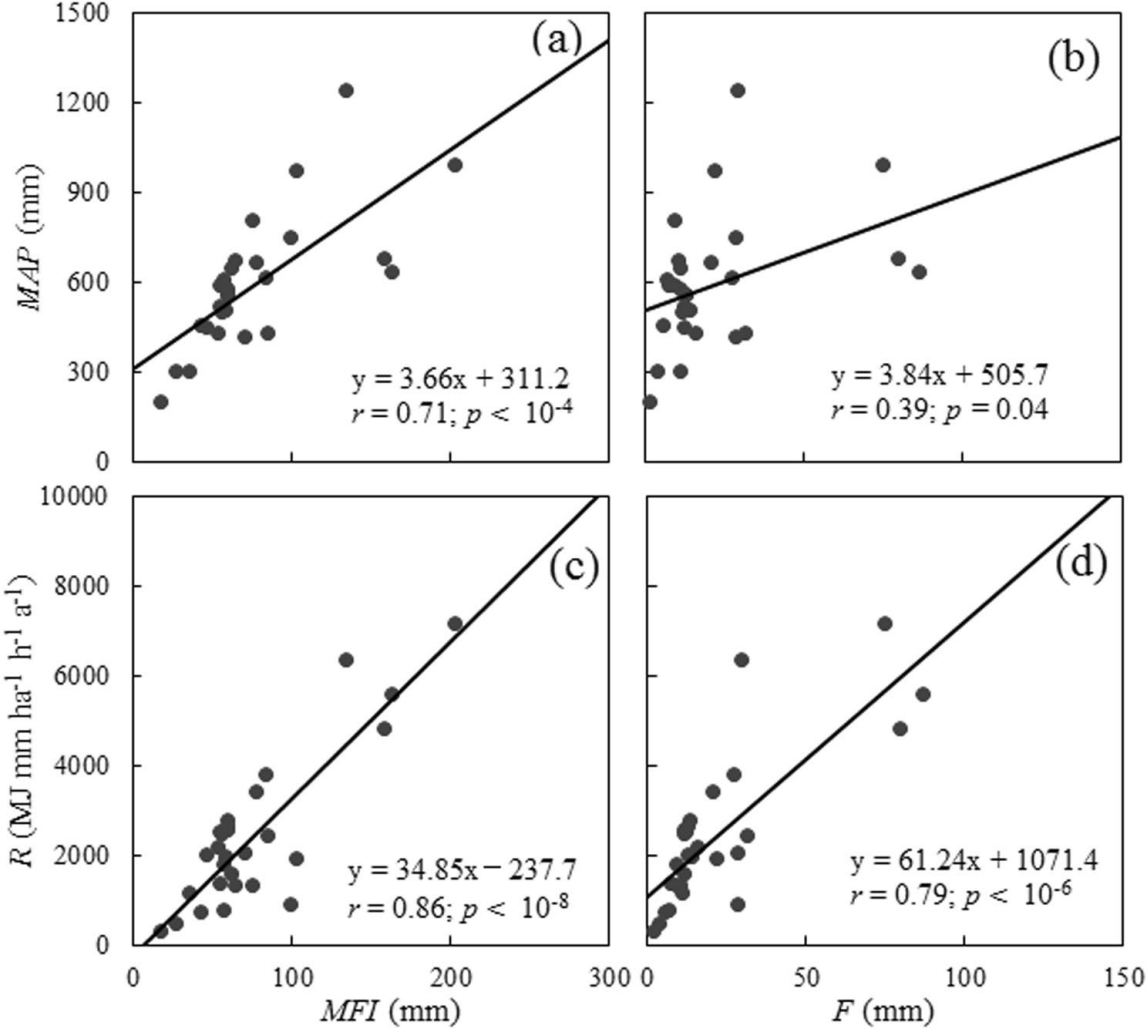
Correlations between the *MFI* and *F,* and *MAP* and *R.*

Among the monthly proxies, *PR*_4_, *PR*_5_, and *PR*_7_ are based on the modified Fournier index (*MFI*), whereas *PR*_6_ is based on the Fournier index (*F*) (Table 1). Both indices have been widely applied to estimate the *R*-factor^31–34^. Renard et al.^10^ and Yue et al. (2014) found that the *MFI* was stronger correlated with *MAP* than the *F*. Arnoldus^35^ and Hernando and Romana^36^ reported a stronger correlation between the *MFI* and *R* than between the *F* and *R*. The Australian data we used agree well with the observations above. Although the *MFI* and *F* were both highly correlated with *MAP* and *R* (Fig. 3), Meng’s test indicated that the *MFI* was stronger correlated with both *MAP* and *R* (*p* < 0.048). The three *MFI*-based monthly proxies, *PR*_4_, *PR*_5_, and *PR*_7_, even showed a stronger correlation with *R* than *PR*_6_ (Eq. (14)), a daily proxy for the *R*-factor. Hence, we concluded that the *MFI* is superior to the *F* in developing a proxy for the *R*-factor. The t-test of the regression equation of *PR*_3_–*PR*_8_ indicated that the coefficients of the equations were significantly unequal to 1. Table 3 shows that directly using the monthly *PR*s to estimate the *R*-factor produced large errors. Therefore, when only monthly rainfall data are available, it is better to use the revised *PR*_5_ and *PR*_7_ to predict the *R*-factor.

Among the daily proxies, *PR*_14_ and *PR*_15_ were developed in Australia, and their correlation with *R* ranked first among the 15 proxies. *PR*_11_ and *PR*_13_, two Chinese proxies, ranked second in terms of their correlation with *R*. The reason for the superior performance of *PR*_14_ and *PR*_15_ may not simply be due to the fact that they were established using Australian data. Capturing the periodic variation within a year, the model developed by Yu and Rosewell^37^ (the original version of *PR*_14_ and *PR*_15_) performed best among the 8 *R*-proxies in northeastern Spain^7^.

The t-test of the regression equation of *PR*_10_–*PR*_13_ indicated that the coefficient and the constant were equal to 1 (*p* > 0.06) and 0 (*p* > 0.33), respectively. Table 3 illustrates that no remarkable improvement was observed in the deviation of the *R* value when estimated by the revised equations. Consequently, *PR*_10_–*PR*_13_ can be used to estimate the *R* value. The coefficient and constant term of *PR*_9_ were significantly unequal to 1 and 0, respectively. The revised *PR*_9_ was recommended when estimating the *R*-factor in Australia. The coefficient of *PR*_14_ was significantly equal to 1 (*p* = 0.87) and the constant was notably greater than 0 (*p* = 0.02), which indicated that the equation for *PR*_14_ in Fig. 1k probably leads to a large deviation in estimating *R* values for dry areas. The constant term of *PR*_15_ was equal to 0 (*p* = 0.45) and the coefficient was significantly greater than 1 (*p* = 0.00), which indicated that *PR*_15_ underestimated the *R* value. *PR*_15_ was established using the RUSLE model, which may have led to this result. Nearing et al. (2017) concluded that the RUSLE underestimated 14%, compared with the RUSLE2 and the USLE, in estimating the *R*-factor. This result coincided with the coefficient of 0.19, which is shown in Fig. 1l. We suggest the use of revised *PR*_14_ and *PR*_15_ as surrogates of the *R*-factor when daily rainfall data are available. However, with *MRE*s of 26.1% and 23.6%, respectively (Fig. 1i,j), the accuracy remains satisfactory while using *PR*_11_ and *PR*_13_ as a surrogate of the *R*-factor.

## Conclusion

This study statistically evaluated 15 developed *PR* using rainfall data from Australia. All proxies were found to be significantly correlated with *R* (*p* < 0.004), and thus can be used to predict the *R*-factor by means of linear calibration models. Meng’s test ranked the correlations between *R* and *PR* (see Eq. (14)), indicating that the daily proxies were stronger correlated with *R* than the monthly ones, which in turn were stronger correlated than the yearly ones. Furthermore, the t-test of the regression equations showed that five proxies (*PR*_1_, *PR*_10_, *PR*_11_, *PR*_12_, and *PR*_13_) were statistically equal to the *R*-factor, implying that they can be directly used as an *R* surrogate without calibration. A direct estimation of the *R*-factor using *PR*s produced an *MRE* between 18.3 and 153.0%, with a mean of 50.0%. The linear calibrations produced an *MRE* ranging from 13.1 to 77.4%, with a mean of 36%.

Based on the statistical evaluations above, we recommended proxies for instances where only daily, monthly, or yearly rainfall data are available. Our rainfall data were collected from varying climate zones (from tropical to temperate, and from humid to semi-arid and arid). Hence, although future work in other regions is needed, our results may be applicable to other regions around the world.

## Abbreviations

R: Rainfall erosivity calculated by RUSLE2, MJ mm ha^−^^1^ h^−^^1^ a^−^^1^
PR: Rainfall erosivity calculated by proxy model, proxy for R, MJ mm ha^−^^1^ h^−^^1^ a^−^^1^
MAP: Mean annual precipitation, mm
MRE: Mean relative error, %
RMSE: Root mean square error, MJ mm ha^−^^1^ h^−^^1^ a^−^^1^
NSE: Nash–Sutcliffe efficiency coefficient
MFI: Modified Fournier index, mm
F: Fournier index, mm
