# Author Correction: Statistical evaluation of proxies for estimating the rainfall erosivity factor

**DOI:** 10.1038/s41598-022-19412-0

**Published:** 2022-09-12

**Authors:** Xiaoqing Ma, Mingguo Zheng

**Affiliations:** 1 National-Regional Joint Engineering Research Center for Soil Pollution Control and Remediation in South China, Guangdong Key Laboratory of Integrated Agro-environmental Pollution Control and Management, Guangdong Engineering Research Center for Non-point Source Pollution Control, Institute of Eco-environmental and Soil Sciences, Guangdong Academy of Sciences, Guangzhou, 510650 People’s Republic of China; 2 Key Laboratory of Water Cycle and Related Land Surface Processes, Institute of Geographic Sciences & Natural Resources Research, Chinese Academic of Sciences, Beijing, 100101 People’s Republic of China; 3 College of Resources and Environment, University of Chinese Academy of Sciences, Beijing, 100101 People’s Republic of China; 4 International Institute of Soil and Water Conservation, Meizhou, 514000 People’s Republic of China

Correction to: *Scientific Reports* 10.1038/s41598-022-15271-x, published online July 2022

The Funding section in the original version of this article was omitted.

The Funding section now reads: "The study was financially supported by the Guangdong Foundation for Program of Science and Technology Research (2020B1111530001), GuangDong Basic and Applied Basic Research Foundation (2021A1515011552), Guangdong Provincial Science and Technology Project (2021B1212050019), Meizhou Science and Technology Plan Project (2020B0204001) and GDAS' Project of Science and Technology Development (2022GDASZH-2022010203)."

The original Article has been corrected.

